# Effects of vision on energy expenditure and kinematics during level walking

**DOI:** 10.1007/s00421-022-04914-6

**Published:** 2022-03-02

**Authors:** Ola Eiken, Igor B. Mekjavic, Jan Babič, Ulf Danielsson, Magnus Hallberg, Stylianos N. Kounalakis

**Affiliations:** 1 Division of Environmental Physiology, School of Chemistry, Biotechnology and Health, KTH Royal Institute of Technology, Berzelius v. 13, 171 65 Stockholm, Sweden; 2 Department of Automation, Robotics and Biocybernetics, Jožef Stefan Institute, Jamova 39, 1000 Ljubljana, Slovenia; 3 The Land Warfare Centre, Swedish Armed Forces, Kvarn 200, 590 28 Borensberg, Sweden; 4 Department of Physical and Cultural Education, Evelpidon Hellenic Army Academy, Vari, Greece

**Keywords:** Blindfolding, Centre of mass, Gait kinematics, Level walking, Oxygen consumption, Visual cues

## Abstract

**Purpose:**

We have previously observed substantially higher oxygen uptake in soldiers walking on terrain at night than when performing the same walk in bright daylight. The aims of the present study were to investigate the influence of vision on mechanical efficiency during slow, horizontal, constant-speed walking, and to determine whether any vision influence is modified by load carriage.

**Methods:**

Each subject (*n* = 15) walked (3.3 km/h) for 10 min on a treadmill in four different conditions: (1) full vision, no carried load, (2) no vision, no carried load, (3) full vision with a 25.5-kg rucksack, (4) no vision with a 25.5-kg rucksack.

**Results:**

Oxygen uptake was 0.94 ± 0.12 l/min in condition (1), 1.15 ± 0.20 l/min in (2), 1.15 ± 0.12 l/min in (3) and 1.35 ± 0.19 l/min in (4). Thus, lack of vision increased oxygen uptake by about 19%. Analyses of movement pattern, by use of optical markers attached to the limbs and torso, revealed considerably shorter step length (12 and 10%) in the no vision (2 and 4) than full vision conditions (1 and 3). No vision conditions (2 and 4) increased step width by 6 and 6%, and increased vertical foot clearance by 20 and 16% compared to full vision conditions (1 and 3).

**Conclusion:**

The results suggest that vision has a marked influence on mechanical efficiency even during entrained, repetitive movements performed on an obstacle-free horizontal surface under highly predictable conditions.

## Introduction

The present study concerns effects of vision on energy expenditure during slow constant-pace walking. Army soldiers commonly conduct foot-borne operations that include heavy load carriage at night. Typically, the reasons to perform such operations at night are tactical considerations, but during missions in hot climates, another reason may be to reduce heat strain. However, in conjunction with training exercises, we have occasionally observed substantially higher oxygen uptake, and hence endogenous heat production, in soldiers walking on terrain at night whilst using night-vision goggles, than when performing the same task in bright daylight (unpublished observations). Several factors might have contributed to the increased oxygen uptake. For instance, it is conceivable that under conditions of restricted peripheral vision, soldiers walk with higher foot clearance from the ground to reduce the risk of tripping (cf. Begg et al. 2007). Other alterations of the movement pattern, associated with limited visual cues, including shorter step length, increased step width (Bauby and Kuo 2000) and reduced postural stability (Logan et al. 2010), might also contribute to an increased energy expenditure during nighttime operations. There is evidence to support the notions that during level walking, healthy individuals tend to automatically adjust their movement pattern so as to minimize the energy expenditure (Waters et al. 1988) and that vision may play a key role in such adjustments (Reynolds and Day 2005). Likewise, at preset walking velocity, individuals appear to adjust their step length (Zarrugh et al. 1974; Umberger and Martin 2007) and width (Donelan et al. 2001) as well as the vertical centre of mass displacement (Gordon et al. 2009) to minimal energy cost.

Thus, the primary aim was to investigate if lack of vision might increase energy expenditure whilst walking at slow and steady pace under highly predictable conditions, i.e. without any changes in terrain or obstacles on the ground. The slow and obstacle-free walking task was chosen as a safety precaution. In addition, we reasoned that if lack of vision increases oxygen uptake even in such a simple and predictable walking task, then increased energy expenditure may constitute a salient feature during foot-borne movements in darkness. Since vision is important for postural stability during walking (Logan et al. 2010), a secondary aim was to investigate whether preserved vision might be more important for sustaining low energy cost during walking, if the mass and centre of mass of the individual are acutely changed by adding carried load. Accordingly, we measured oxygen consumption and kinematics in individuals walking with full vision or blindfolded on a horizontal treadmill at steady slow speed, with or without carrying a heavy rucksack.

## Materials and methods

### Subjects

Fifteen healthy men participated as test subjects. Their mean ± SD age, stature, and body mass were 29.0 ± 10.9 years, 176.5 ± 5.5 cm and 76.9 ± 13.1 kg, respectively. The subjects were instructed not to engage in any strenuous physical activity during 24 h preceding the experiments and to refrain from caffeine and nicotine on the day of the experiments. Following familiarization with the protocol and methods, each subject gave his informed consent to participate in the study. The protocol was approved by the National Committee for Medical Ethics at the Ministry of Health (Republic of Slovenia) and conformed to the declaration of Helsinki.

### Study outline

Each subject walked on a treadmill (Woodway, PPS Med, USA) at a constant velocity of 3.3 km/h for 10 min in four different conditions:

1. full vision and no carried load,
2. no vision and no carried load,
3. full vision carrying a 25.5-kg rucksack,
4. no vision carrying a 25.5-kg rucksack.

Among subjects, the four trials were performed in an alternating counter-balanced order, each trial being interspersed by a 10-min period, during which the subject was resting motionless in a seated, upright position. For safety reasons, the subject wore a loosely fitting harness, attached at the ceiling by a strap, and the treadmill was provided with handle bars on both sides as well as in the front and back of the tread belt. Additionally, approximately 20 cm in front of the back-side handle bar, the treadmill was equipped with a laser beam, which, if broken, activated an acoustic signal alerting the subject that he was approaching the rear border of the treadmill walking belt.

### Methods and procedures

Before the experiments, the subjects went through a 45-min standardized training procedure, during which they walked with full vision as well as blindfolded on the treadmill (3.3 km/h), getting acquainted with the pace, the handle bars, the laser-beam warning mechanism and the rucksack. During the experiment, the subject wore shorts, t-shirt, long socks and hiking boots (1.3 kg). After donning the harness, he was equipped with 15 reflective markers, taped on the top of the head, and bilaterally on the following locations: the fifth metatarsal joint, the lateral malleolus, the lateral condyle of the tibia, the iliac crest, the dorsal side of the wrist, the lateral epicondyle of the humerus and the greater tuberosity of the humerus. Four more markers were placed at the four corners of the treadmill. Three-dimensional marker trajectory data were collected using a six-camera motion capturing system (SMART-e 600, zFlo, Inc. Lexington, MA), during two 1-min periods in each 10-min trial, namely, min 3–4 (Start) and 8–9 (End) of the trial. All kinematic variables, except for cadence (see “Analysis” section), were calculated as means for each gait cycle (i.e. left and right leg) and then averaged for the entire sampling period.

Heart rate (HR) was monitored continuously using a carry-on system (Polar S-810, Finland). After the positioning of the reflective markers, the subject was equipped with a mobile standalone metabolic cart system (total weight of ~ 1.5 kg; K_4_b^2^, Cosmed, Italy) for measurement of oxygen uptake ($\dot{V}{\text{O}}_{2}$) and expired minute ventilation ($\dot{V}{\text{E}}$); $\dot{V}{\text{O}}_{2}$ and $\dot{V}{\text{E}}$ were measured continuously throughout each treadmill trial. The O_2_ and CO_2_ analyzers of the metabolic cart were calibrated with two different gas mixtures and the flow meter with a 3-L syringe, before each experiment. A black velvet cloth was used for blindfolding. Care was taken to ensure that there was no leakage of light through the blindfold. A standard canvas rucksack with padded, adjustable shoulder and waist belts was used. The rucksack was filled with clothing, shoes and boots, so that the weight (25.5 kg) was evenly distributed in the bag; its weight was measured before the first experiment.

The environmental conditions were kept similar in all trials. Thus, the ambient temperature was 21.0 ± 0.4 °C, and the relative humidity 51 ± 0.5%.

### Analysis

A custom-made computer program, based on MATLAB (MathWorks Inc, USA), was developed to determine all kinematic variables from the motion capturing data. The computer identifications of reflective markers during the walking trials were always checked manually, and if need be, corrected/intrapolated, before allowing the program to estimate the following kinematic variables:

- Stride length: the antero-posterior distance between the sequential points of initial contact by the same foot.
- Cadence: the step rate per minute.
- Step width: the medio-lateral distance between the midlines of the feet.
- Foot clearance: the minimal distance of the feet from the treadmill surface during the swing phase of the gait cycle.
- Hip vertical amplitude: the maximal vertical displacement of the iliac crest, with the treadmill surface as the reference, during the gait cycle.

A two-way ANOVA with repeated measures was used to define the effects of weight bearing and vision on the variables measured (absolute values). A Bonferonni post hoc test was employed to assign the specific differences in the analysis of variance in case of a significant main effect. For the cardiorespiratory variables, the last 8 min of each treadmill trial was included in the analyses, and for the kinematic variables, both 1-min sample periods (see above) were included in the analyses. Values are presented as means (SD). The significance level was set at 0.01. Note that the statistical analyses are based on absolute values, which are also presented in figures and table, whereas, for comparison, relative values (% changes based on absolute means) are given in the narrative of “Results”.

## Results

All experiments were performed without any adverse events. Several of the subjects were, on one or a few occasions, alerted by the acoustic warning signal that they were approaching the rear end of the tread belt, whereas on no occasion did any of the subjects make use of the handle bars or safety harness.

### Cardiorespiratory responses

**Fig. 1:**
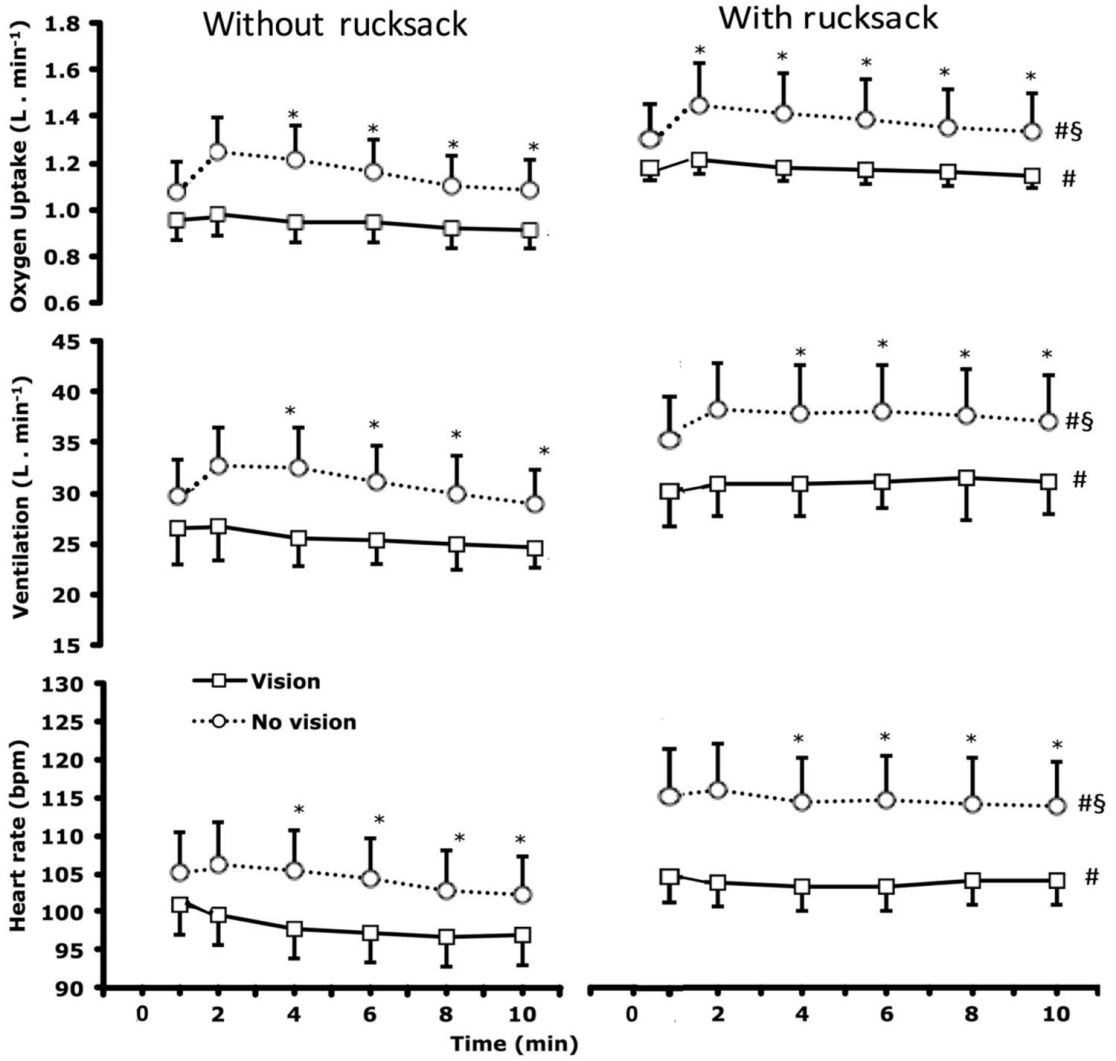
Oxygen uptake (upper panels), pulmonary minute ventilation (middle panels) and heart rate (lower panels) during 10-min treadmill walking, with and without vision and without (left panel) and with (right panel) a 25.5-kg rucksack. Values are mean ± SD of the preceding time period. Statistical analyses are based on the last four values (min 2–10) of each trial; *n* = 15. *Difference between vision and corresponding no vision condition (*p* < 0.01). ^#^Difference from condition without rucksack and with vision (*p* < 0.01). ^§^Difference from condition without rucksack and without vision (*p* < 0.01)

The $\dot{V}{\text{O}}_{2}$ $\dot{V}{\text{E}}$, and HR responses are presented in Fig. 1, and, along with some background cardiorespiratory variables, in Table 1. It can be seen that blindfolding increased $\dot{V}{\text{O}}_{2}$ by 19% [*F*(1,14) = 22.4, *p* < 0.01; Fig. 1, Table 1]. Likewise, blindfolding increased $\dot{V}{\text{E}}$ by 20% [*F*(1,14) = 26.3, *p* < 0.01] and HR by 8% [*F*(1,14) = 17.4, *p* = 0.01] (Fig. 1, Table 1).

**Table 1:** Cardiorespiratory responses during 10 min of constant-pace walking with or without vision and with or without a 25.5-kg rucksack

	Without rucksack		With rucksack	
	Vision	No Vision	Vision	No Vision
$\dot{V}{\text{O}}_{2}$ (ml min^−1^)	940 ± 127	1145 ± 196*	1154 ± 125#	1345 ± 188*#
$\dot{V}{\text{O}}_{2}$ (ml kg^−1^ min^−1^)	12.3 ± 1.3	15.2 ± 3.4*	15.2 ± 2.1#	17.8 ± 3.3*#
V̇CO_2_ (ml min^−1^)	729 ± 114	892 ± 193*	882 ± 129#	1037 ± 184*#
$\dot{V}{\text{E}}$ (l min^−1^)	25.6 ± 2.8	30.8 ± 5.0*	30.0 ± 3.1#	36.1 ± 5.4*#
HR (beats min^−1^)	98 ± 12	104 ± 15*	103 ± 11#	113 ± 16*#
RER	0.78 ± 0.05	0.78 ± 0.06	0.76 ± 0.06	0.77 ± 0.06
Rf (breaths min^−1^)	24 ± 4	28 ± 4*	27 ± 5#	33 ± 6*#
Oxygen pulse (ml beat^−1^)	9.7 ± 1.8	11.0 ± 1.4*	11.3 ± 1.8#	11.9 ± 1.7*#

Values are means ± SD, *n* = 15

$\dot{V}{\text{O}}_{2}$ oxygen consumption, $\dot{V}{\text{E}}$ minute ventilation, *HR* heart rate, *RER* respiratory exchange ratio, *Rf* respiratory frequency. Significant differences derived from two-way ANOVA

*Difference compared to the corresponding vision condition, *p* < 0.001

^#^Difference compared to the corresponding condition without rucksack, *p* < 0.001

Carrying the rucksack increased $\dot{V}{\text{O}}_{2}$ by 20% [*F*(1,14) = 233.2, *p* < 0.01], $\dot{V}{\text{E}}$ by 17% [*F*(1,14) = 93.1, *p* < 0.01] and HR by 7% [*F*(1,14) = 36.3, *p* < 0.01] (Fig. 1, Table 1), but similarly whether or not the subjects were blindfolded [interaction effects for $\dot{V}{\text{O}}_{2}$: *F*(1,14) = 0.1, *p* = 0.7; for $\dot{V}{\text{E}}$: *F*(1,14) = 0.9, *p* = 0.3; and HR: *F*(1,14) = 2.7, *p* = 0.1).

No significant changes over time were observed in the cardiorespiratory responses (*F* values between 0.1 and 2.7, *p* > 0.05).

### Kinematic variables

**Fig. 2:**
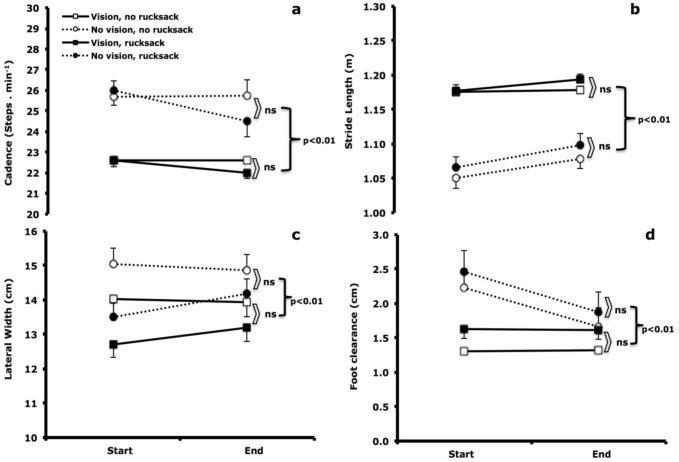
Walking cadence (**a**), stride length (**b**), step width (**c**) and foot clearance (**d**) at the beginning (min 3–4; Start) and before the end of the 10 min walking trials (min 8–9; End). Values are mean ± SD, *n* = 15. *ns* no significant differences

Blindfolding increased walking cadence by 11% [*F*(1,11) = 14.9 *p* < 0.01; Fig. 2). Likewise, blindfolding increased the step width by 6% [*F*(1,11) = 9.5, *p* = 0.01] and the vertical foot clearance by 18% [*F*(1,11) = 9.4, *p* = 0.01] (Fig. 2). Blindfolding shortened stride length by 11% [*F*(1,11) = 32.2, *p* < 0.01; Fig. 2). Lastly, the hip vertical amplitude was not affected by the lack of vision [values ranged between 3.8 and 4.6; *F*(1,11) = 0.1, *p* = 0.9].

The 25.5-kg rucksack had no effect on any of the kinematic variables (no significant main effects of load carriage, F values between 1.2 and 3.3, *p* > 0.05) and no significant interaction between load carriage and vision was observed (interaction effects for cadence: *F*(1,11) = 0.9, *p* = 0.3; for step width: *F*(1,11) = 0.4, *p* = 0.5; for foot clearance: *F*(1,11) = 0.1, *p* = 0.7; and for hip vertical amplitude: *F*(1,11) = 0.1, *p* = 0.9 Fig. 2).

No significant changes over time were observed in the walking cadence [*F*(1,11) = 0.9, *p* = 0.3], the step width [*F*(1,11) = 0.4, *p* = 0.5] or the stride length [*F*(1,11) = 2.7, *p* = 0.1], whereas the foot clearance was reduced during the course of the 10-min treadmill walk by 4% [*F*(1,11) = 21, *p* < 0.01; interaction effect: *F*(1,11) = 0.1, *p* = 0.7; Fig. 2D].

## Discussion

The aims of the present study were to investigate the influence of vision on energy expenditure and kinematics during slow, horizontal, constant-speed walking; and whether preserved vision is more important for minimizing energy expenditure if the walker is carrying a heavy backpack. The results demonstrated that lack of vision increased oxygen uptake by 19%, which incidentally corresponded approximately to the added oxygen consumption induced by carrying the 25.5-kg rucksack with vision. Lack of vision increased energy expenditure similarly whether or not the walker was carrying a 25.5-kg rucksack. Lack of vision reduced stride length and hence, increased step cadence and increased foot clearance as well as step width. Gait kinematics were not affected by carrying the rucksack at the slow, pre-determined treadmill speed.

Our observation that the blindfolding increased oxygen uptake during the treadmill walking by 19% is in agreement with the finding that blind individuals exhibited a 25% higher oxygen uptake during level treadmill walking at 4.8 km/h than did their sighted controls (Kobberling and Jankowski 1989). Presumably, the changes in gait kinematics resulting in increased energy expenditure in unsighted conditions are primarily induced to secure postural stability (cf Logan et al. 2010). Similar increments in energy expenditure in acutely blindfolded subjects as in chronically blind individuals (Kobberling and Jankowski 1989) may also suggest that the tendency of vision to secure balance at the expense of mechanical efficiency during walking is not readily compensated for by training. To what extent the foveal and peripheral vision, respectively, contributes to such optimization of balance and energy demand remains to be settled. Judging by our aforementioned observations that oxygen uptake during walking may be higher whilst using night-vision googles than with full vision, and by the finding that, during level walking, the gait pattern is affected by restriction of the visual field (Graci et al. 2009), it appears that peripheral visual cues, known to be critical for spatial orientation (for review see Rollin Stott and Benson 2016), may play a key role to minimize the metabolic cost of walking.

The notion that in healthy individuals, the preferred manner of walking is gauged so as to minimize the energy expenditure is supported by a plethora of studies (for review see Saibene and Minetti 2003). It appears that, during level walking, the choice of stride length is a critical factor for such minimization (Zarrugh et al. 1974; Umberger and Martin 2007). It can thus be presumed that the considerable shortening of the stride length during the present non-vision trials contributed to the increased energy expenditure in these conditions. Judging by previous studies (Umberger and Martin 2007), it appears that about half of the present increase in metabolic energy expenditure in the non-vision trials can be attributed to the reduced stride length/increased cadence. In line with present findings, it has been shown that the preferred stride length is considerably shorter in late blind, and particularly in congenitally blind, than in sighted controls (Nakamura 1997). In the present blindfolded walks, also the increased vertical clearance of the feet presumably contributed substantially to the increased metabolic energy expenditure (Faraji et al. 2018), whereas the minute increase in step width likely had only a marginal effect on energy expenditure (Donelan et al. 2001; Faraji et al. 2018).

It is tempting to speculate that the oxygen demanding alterations of gait kinematics during the present blindfolded trials were induced, consciously or subconsciously, to secure postural stability (cf Logan et al. 2010). Notwithstanding, the craniad shift of centre of mass (i.e. body mass + carried mass), induced by carrying the 25.5-kg rucksack, did not aggravate the alterations in gait pattern in the blindfolded subjects. Thus, as expected in the sighted subjects, the addition of carried load increased oxygen uptake (cf Boffey et al. 2019), but did not alter the present gait variables. That the carried load did not affect stride length, cadence or step width appears to be in line with the majority of previous publications (for review see Walsh and Low 2021). Blindfolding increased oxygen uptake, reduced stride length and increased step width and vertical clearance of the feet similarly—whether or not the subject was carrying a rucksack (Table 1). Thus, it appears that during slow, constant-speed level walking, vision, but not centre of mass, is critical for minimizing energy expenditure.

### Methodological considerations

The present experiments were performed on a treadmill with the subject walking at a slow constant speed. On the one hand, this ensured obstacle-free, highly predictable walking conditions that were identical between subjects and trials. On the other hand, the waking pace was preset (enforced), and it therefore cannot be excluded that under more realistic conditions, for instance whilst walking on a horizontal road, the blindfolded subjects might have chosen a different, less oxygen consuming pace. Notably, at a given pace between 2.9 and 5.9 km/h, the metabolic demand has been found identical whether the walking is conducted on a treadmill or on a horizontal hard surface (Ralston 1960).

Furthermore, considering the original context of the present study, namely nighttime patrol missions, neither obstacle-free horizontal terrain nor a complete lack of visual cues appear very realistic. Thus, the present results need to be supplemented by controlled experiments regarding the effects of night-vision goggles and differing terrain on energy expenditure during nighttime walking.

## Conclusion

In conclusion, blindfolding increased oxygen consumption during slow, constant-speed walking on a horizontal treadmill by 17% and 22%, respectively, with and without carrying a 25.5-kg rucksack. This suggests that vision has a marked influence on mechanical efficiency even during entrained, repetitive movements performed on an obstacle-free horizontal surface under highly predictable conditions.

## Abbreviations

HR: Heart rate
$\dot{V}{\text{O}}_{{2}}$: Minute oxygen uptake
$$\dot{V}_{{\text{E}}}$$: Minute ventilation
ANOVA: Analysis of variance
